# Lagrangian dynamical geography of the Gulf of Mexico

**DOI:** 10.1038/s41598-017-07177-w

**Published:** 2017-08-01

**Authors:** P. Miron, F. J. Beron-Vera, M. J. Olascoaga, J. Sheinbaum, P. Pérez-Brunius, G. Froyland

**Affiliations:** 1 Department of Atmospheric Sciences, Rosenstiel School of Marine and Atmospheric Science, University of Miami, Miami, Florida USA; 2 Department of Ocean Sciences, Rosenstiel School of Marine and Atmospheric Science, University of Miami, Miami, Florida USA; 3 Departamento de Oceanografía Física, Centro de Investigación Cientfica y Educación Superior de Ensenada, Ensenada, Baja California Mexico; 4 School of Mathematics and Statistics, University of New South Wales, Sydney, Australia

## Abstract

We construct a Markov-chain representation of the surface-ocean Lagrangian dynamics in a region occupied by the Gulf of Mexico (GoM) and adjacent portions of the Caribbean Sea and North Atlantic using satellite-tracked drifter trajectory data, the largest collection so far considered. From the analysis of the eigenvectors of the transition matrix associated with the chain, we identify almost-invariant attracting sets and their basins of attraction. With this information we decompose the GoM’s geography into weakly dynamically interacting provinces, which constrain the connectivity between distant locations within the GoM. Offshore oil exploration, oil spill contingency planning, and fish larval connectivity assessment are among the many activities that can benefit from the dynamical information carried in the geography constructed here.

## Introduction

Over the past few decades a number of satellite-tracked surface drifting buoys have surveyed the Gulf of Mexico (GoM). Much insight into the GoM’s surface-ocean Lagrangian dynamics has been gained from the analysis of different subsets of the collected drifter data. A large body of the work done was dedicated to study relative dispersion statistics using pairs of drifter trajectories and their velocities in an attempt to deduce the shape of the kinetic energy wavenumber spectrum^1–5^. Other work employed drifter trajectory data to assess the significance of transport patterns detected from altimetry-derived velocity using nonlinear dynamics tools^6–8^. Additional work was more concerned with making practical use of the drifter data through assimilating drifter velocities into ocean general circulation models^9^ and blending these velocities with altimetry-derived velocities to improve near-real-time synoptic estimates of ocean currents^10^. Descriptive studies were also reported highlighting preferred synoptic pathway patterns^11–14^.

The number of drifters that have surveyed the GoM is large enough that a global characterization of the GoM’s Lagrangian dynamics can be sought. This is indeed possible thanks to probabilistic tools which enable sketching absorbing and almost-invariant sets in the phase space of a nonlinear dynamical system^15–17^. The relevant nonlinear dynamics here are the Lagrangian dynamics, which can be appropriately discretized using those tools into a Markov chain described by a matrix of transitional probabilities of moving between states of the chain. Inspection of the eigenvectors of the transition matrix^18^ enables localization of regions of the flow where trajectories converge in forward time as well as the regions where those trajectories originate from (i.e., their backward-time basins of attraction), thereby determining the connectivity between separated locations in the flow domain. This is conceptually very different than the traditional approach to population connectivity in marine systems, wherein transition matrices are constructed based on ad-hoc partitions of the flow domain into putative spawning and recruitment areas^19^. The eigenvector method of Froyland *et al*.^18^ we employ here also differs from the recent flow network approach^20, 21^ as the former analyzes time-asymptotic aspects of the dynamics through spectral information from the generating Markov chain, while the latter computes various graph-based quantities for finite-time durations to study flow dynamics. We note that Froyland’s *et al*.^18^ approach is also subtly different from the eigenvector method of Froyland *et al*.^22^, where structures that are approximately fixed over a finite time duration were extracted. Although attracting regions may be small and trap trajectories for long periods of time before eventually exiting and thus constituting almost-invariant regions, if their basins of attraction are large, they can exert great influence on the global Lagrangian dynamics. Decompositions of the surface-ocean flow into almost-invariant sets form the basis of a *Lagrangian dynamical geography*, where the boundaries between basins are determined by the Lagrangian circulation itself, instead of arbitrary geographical divisions.

Our goal in this paper is to construct such a Lagrangian dynamical geography for the GoM, which carries useful information for guiding activities dealing with hampering and/or palliating the effects of an oil spill or a harmful algal bloom or supporting stock assessment efforts and management decisions for fishing regulations, just to cite some examples.

## Results

**Figure 1:**
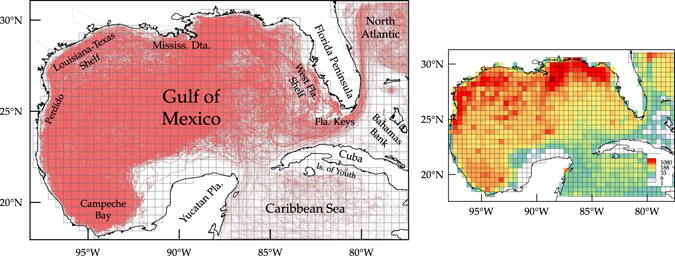
(left) “Spaghetti” plot of all daily satellite-tracked drifter trajectories describing the surface-ocean Lagrangian dynamics in the extended Gulf of Mexico (eGoM) domain. The finite grid used to construct a Markov-chain representation of the dynamics is shown in black. (right) Number of drifters per grid bin independent of the day over 1994–2016 subjected to a fourth-root transformation. Figures constructed using Matlab R2017a (http://www.mathworks.com/) and Tecplot 360 2016 R2 (http://www.tecplot.com/).

The left panel of Fig. 1 shows all 3207 daily drifter trajectories (in red) available to us over 1994–2016 for describing the surface-ocean Lagrangian dynamics in the domain of interest. The domain, referred to herein as extended GoM (eGoM), includes the GoM itself and small adjacent portions of the Caribbean Sea and North Atlantic. The “spaghetti” plot shown in the figure reveals that the drifters sample most of the domain (about 3 drifters are found per km^2^ on average ignoring time). Exceptions are relatively small regions on the Yucatan Shelf, south of Cuba, and the Bahamas Bank, which have never been visited by any drifters. As described in some depth in Materials, the drifters differ in design from experiment to experiment, so some variations in their Lagrangian properties can be expected^23^. For the purposes of this work, these variations were ignored.

### Construction of the Markov-chain model

In order to apply the eigenvector method, outlined in Methods, we proceeded to discretize the Lagrangian dynamics as represented by the drifter motion as follows. We first laid down on the eGoM a grid of *N* = 1500 square bins of (roughly) 50-km side as shown (in black) in the left panel of Fig. 1. While we did not perform optimizations of any kind, this grid resolution resulted in a reasonable individual bin coverage as shown in the drifter density plot in the right panel of Fig. 1. Ignoring time, there are 246 drifters on average per bin, with as many as 4266 drifters in some bins and a few (108) empty bins.

We then constructed, according to (1), the *N* × *N* matrix *P* of transitional probabilities of the drifters to move between bins of the grid (i.e., states of the Markov chain defined by *P*) using 2-day-long trajectory pieces, which may begin on any day. Neglecting the start day corresponds to assuming that the Lagrangian dynamics are statistically stationary. This is clearly a strong assumption, which is commonly made in relative dispersion analyses in connection with turbulence phenomenology studies^24^. This assumption may be more easily justified when the focus is on long-time behavior, as is the case of this work and earlier related works^18, 25, 26^. On the other hand, 2-day-long trajectory pieces are long enough to enable interbin communication in most cases. Furthermore, 2 days is larger than the typical Lagrangian decorrelation timescale near the ocean surface, estimated to be of about 1 day^27^. Thus there is negligible memory farther than 2 days into the past, and so the Markov assumption is approximately satisfied.

An analysis of the sensitivity to transition time changes and data reductions is presented in Appendix A of the Supplementary Information. This reveals that the results discussed below do not depend on the above specific choices. Independent of these choices, a few bins were always found to be initially empty, which had to be excluded, making *P* always slightly deviate from being row-stochastic (some of its rows did not add up to 1 exactly). This required us to row-stochasticize *P* by normalizing each row by its sum, following standard procedures^18^.

### Communication within the Markov chain

**Figure 2:**
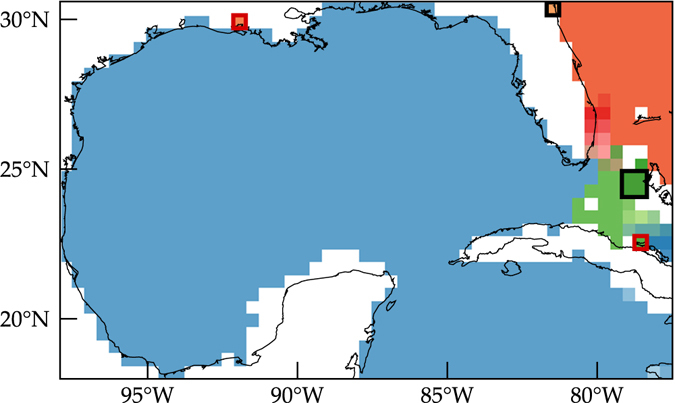
Grouping of the Markov-chain states into classes according to their communication type. Bins indicated with the same color belong to the same class. The are 31 communicating classes covering the eGoM domain. Two of these classes are closed (red boxes) and 2 are both closed and absorbing (black boxes); they do not offer any insight into the Lagrangian dynamics other than mere beaching of drifters. Computations carried using Matlab R2017a (http://www.mathworks.com/) and visualization using Tecplot 360 2016 R2 (http://www.tecplot.com/).

Once the transition matrix *P* was constructed, we moved on to determine the level of communication among the states of the Markov chain. Let $S\subset \{1,\ldots ,N\}$ represent a class of states; the following communication types can be distinguished^28^. The class *S* is said to be communicating if for each pair *i*, *j* ∈ *S* there exists *m* > 0 finite such that (*P* ^*m*^)_*ij*_ > 0 (i.e., there is a positive probability of moving between any pairs of states in the class in a finite number of steps). A communicating class *S* is said to be closed if it has *P*_*ij*_ = 0 for all *i* ∈ *S*, $j\notin S$ (i.e., there is a zero probability of moving out of the class to another state elsewhere in the computational domain). A communicating class *S* is said to be absorbing if given some *m* > 0 finite, (*P* ^*m*^)_*ij*_ > 0 for at least one $i\notin S$, *j* ∈ *S* (i.e., there is a positive probability of at least one external state moving into the class in a finite number of steps). In practice a Markov chain can be viewed as a directed graph with vertices in the graph corresponding to states in the chain, and directed arcs in the graph corresponding to one-step transitions of positive probability. This allows one to apply Tarjan’s algorithm^29^ to assess communication within a chain. Specifically, the Tarjan algorithm takes such a graph as input and produces a partition of the graph’s vertices into the graph’s strongly connected components. A directed graph is strongly connected if there is a path between all pairs of vertices. A strongly connected component of a directed graph is a maximal strongly connected subgraph and by definition also a maximal communicating class of the underlying Markov chain. Applying Tarjan’s algorithm to the Markov chain derived using the drifter trajectory data we found a total of 31 strongly connected components or, equivalently, maximal communicating classes. Each one of these classes is indicated with a different color in Fig. 2. Direct verification further showed that, out of the 31 communication classes revealed by the Tarjan algorithm, there are 2 closed communicating classes, one consisting of a single state and another one made up of four states; the two closed classes are highlighted with red boxes in Fig. 2. Two additional single-state communicating classes (highlighted with black boxes in Fig. 2) were determined by direct verification to be both closed and absorbing.

The closed communicating classes correspond to coastal or near-coastal regions where the drifters initially lying inside do not move or stay within, or initially lying outside get trapped within. Other than reflecting beaching, these classes do not represent any dynamically relevant process. Relevant from a Lagrangian dynamics viewpoint is the large group of communicating classes, which cover almost entirely the eGoM. This group is composed of 2 large classes, one covering the entire GoM and the Caribbean Sea portion of the eGoM (indicated in blue) and the other one spanning a large part of the North Atlantic sector (in orange), and 25 additional smaller classes occupying the rest of the North Atlantic sector (in red and darkgreen tones). Communication among the states of this group is permitted. Communication with states outside of the group is possible too due to the presence of the small absorbing classes.

The presence of the small closed communicating classes implies that the forward evolution of initially arbitrary distributed tracers on the eGoM under the action of the transition matrix *P* will never attain a unique invariant distribution. A tracer initially covering the entire eGoM will nevertheless be supported on these small classes in the long run. The tracer initially inside the regions occupied by the closed classes will remain within at all times, while the tracer outside of these regions will converge, eventually after a very long time, into the regions occupied the absorbing classes. But the dynamically relevant phenomena are transient, occur during earlier times, and are not influenced by the small closed communicating classes, which we have verified by direct computation.

### Forward evolution of a tracer density

**Figure 3:**
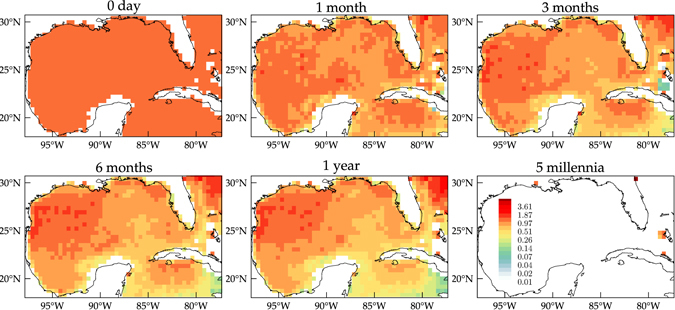
Forward evolution of an initially uniformly distributed tracer under the action of the transition matrix *P*. The tracer concentration is normalized by the mean concentration and subjected to a fourth-root transformation. Computations carried using Matlab R2017a (http://www.mathworks.com/) and visualization using Tecplot 360 2016 R2 (http://www.tecplot.com/).

Figure 3 shows selected snapshots of the forward evolution (of the probability density) of a tracer, initially uniformly distributed over the entire eGoM grid, under the action of *P*, according to (2). It is immaterial to the method whether *P* models purely advective dynamics or a combination of advective and diffusive dynamics^30^. Because *P* was constructed using 2-day-long drifter trajectory pieces, pushing forward the tracer 1 step is equivalent to evolving the tracer in forward time for 2 days. As time advances the tracer distribution loses homogeneity, increasing its density on various regions well separated from one another on the northwestern and eastern sides of the GoM, a region south of Cuba in the Caribbean Sea, and the northeastern side of the North Atlantic portion of the eGoM. These regions show accumulation trends with varied levels of persistence, thereby representing almost limiting invariant distribution regions. Out of the four regions, the region lying in the North Atlantic shows a sustained accumulation trend over several years (15 years or so). This accumulation, however, does not represent any real aspect of the Lagrangian dynamics. Rather, it reflects that the eGoM is closed and the tracer must eventually exit the GoM and Caribbean Sea sectors of the eGoM through the Straits of Florida into the North Atlantic. Considering that the GoM is fully drained when the tracer mass within has decreased by 95%, we estimate a residence timescale for the GoM of about 13 years. Accumulation on the other regions is much more meaningful from a Lagrangian dynamics stand point. Lasting for about 4 years, accumulation in the northwestern side of the GoM on the southern end of the Texas–Louisiana Shelf, more specifically a region lying near the Perdido Foldbelt, implies the existence of a persistent convergent circulation along the coast from the south and north and possibly also a mean westward flow. The accumulation trend on the northern part of the Caribbean Sea south of Cuba is shorter, of a few months, possibly reflecting trapping inside transient closed circulations there or accumulation induced by Ekamn transport associated with trade winds. The eastern side of the GoM over the northern West Florida Shelf and the Florida Keys show a somewhat less persistent accumulation trend, consistent with a relatively rapid flow along the shelfbreak and out of the GoM through the Straits of Florida. As anticipated, the small closed communicating classes do not determine the above accumulation regions or influence their duration trends as it takes very long time (nearly 5 millennia!) for the initially uniform distribution to settle on them.

### Analysis of the Markov chain’s eigenspectum

We can now turn to the application of the eigenvector method, which enables a connection between structure in the eigenvector fields of the transition matrix *P* with (almost) invariant flow regions (or sets) that attract tracer into as well as their corresponding basins of attraction.

First we note that eigenvalue *λ* = 1 of *P* is the largest and further has multiplicity 4, consistent with the Markov chain having 4 closed communicating classes of states. The corresponding left eigenvectors of *P* reveal invariant distributions supported on the small regions occupied by them (i.e., they do not change under the action of *P*) and the right eigenvectors their basins of attraction. The invariant distributions are also limiting distributions, as the forward evolution of a uniform tracer has revealed. As we have noted, they do not represent any relevant aspect of the Lagrangian dynamics other than mere beaching. Thus we do not consider them any further here, but offer a discussion for completeness in Appendix B of the Supplementary Information.

**Figure 4:**
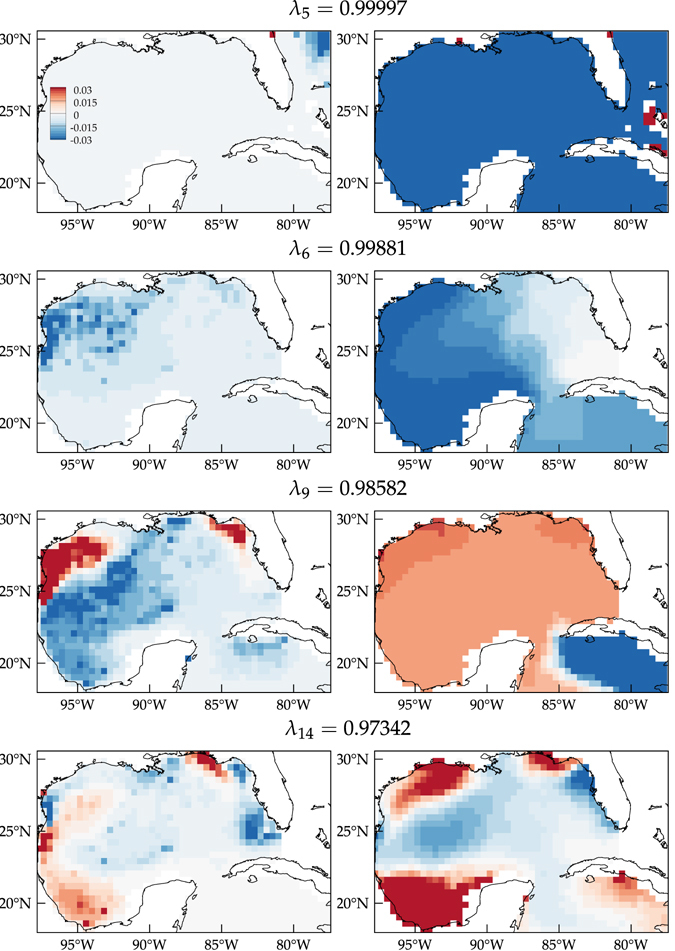
(left) Selected left eigenvector fields of the transition matrix *P* showing the locations of almost-invariant regions of forward-time attraction. (right) Corresponding right eigenvector fields showing the backward-time basins of attraction for the attractors on the left. Computations carried using Matlab R2017a (http://www.mathworks.com/) and visualization using Tecplot 360 2016 R2 (http://www.tecplot.com/).

Dynamically relevant are the larger regions showing shorter but still persistent accumulation trends as the initially uniformly distributed tracer is pushed forward by *P*. These can be revealed by the eigenvector method as they have imprints on the left eigenvectors of *P* with *λ* ≈ 1. Their basins of attraction have their footprints on the corresponding right eigenvectors. Figure 4 shows 4 selected *λ* ≈ 1 left eigenvector fields on the left with the corresponding right eigenvectors on the right. The dominant (*λ* = 0.99997) eigenvectors are shown in the top row. The left eigenvector is supported on the accumulation region on the northeastern corner of the eGoM revealed from direct tracer forward evolution, thereby revealing this region as an attracting set which remains almost-invariant under the action of *P*. As noted above this accumulation reflects the fact that the GoM and Caribbean Sea portions of the eGoM must be evacuated by tracers through the Straits of Florida. Consistent with this, the right eigenvector identifies the basin of attraction of this almost-invariant attracting set with the whole eGoM modulo the small closed communicating class regions. There the right eigenvector is positive and indistinguishably flat.

Before proceeding to the analysis of the other eigenvectors, a few remarks are in order. First, the above large basin of attraction may, and certainly does as we show below, include smaller basins of attraction for other almost-invariant attracting sets contained within. Second, those domains of attraction are weakly dynamically interacting in the sense that they do not have perfect but rather permeable boundaries on the long run, consistent with the fact that *λ* is not exactly unity. Third, because eigenvectors with nearly the same eigenvalue (*λ* ≈ 1) can be linearly combined to form an eigenvector with nearly the same eigenvalue, the smaller almost-invariant attracting sets can also approximately span a larger almost-invariant attracting set through linear combination.

With the above observations in mind, we now proceed to analyze the remaining eigenvectors. The second row of Fig. 4 shows left and right eigenvectors with *λ* = 0.99881, the second largest nonunity eigenvalue. The left eigenvector (left panel) is supported on a region on the northwestern GoM covering mainly the southern part of the Texas–Louisiana Shelf near the Perdido Foldbelt, which coincides with the area revealed from direct tracer evolution that showed the second longest accumulation trend. This region is now identified as an almost-invariant attracting set by the eigenvector method. The right eigenvector (right panel) reveals a partition of the GoM into two halves by a nearly straight boundary roughly connecting the Mississippi River Delta and the easternmost tip of the Yucatan Peninsula. On the western half the right eigenvector takes a nearly constant positive value, identifying this region with the basin of attraction for the almost-invariant attracting set revealed by the left eigenvector.

The *λ* = 0.98582 left and right eigenvectors reveal additional well-defined almost-invariant attracting sets and basins of attraction (Fig. 4, third row). Specifically, a large region on the Texas–Louisiana Shelf, a portion of the northern West Florida Shelf, a domain south of Cuba east of the Island of Youth, and an ample region on the western side of the GoM are revealed as almost-invariant areas of attraction (left panel). The corresponding basins of attraction (right panel) span larger regions including those areas.

The *λ* = 0.97342 left–right eigenvector pair unveils further almost-invariant attracting sets and basins of attraction (Fig. 4, bottom row). Most notably, the Florida keys are identified as an almost-invariant region of attraction by the left eigenvector and the West Florida Shelf as its basin of attraction by the right eigenvector. Likewise, the southwestern portion of the Bay of Campeche is revealed as an almost-invariant attracting set with the corresponding basin of attraction spanning the whole Bay of Campeche.

**Figure 5:**
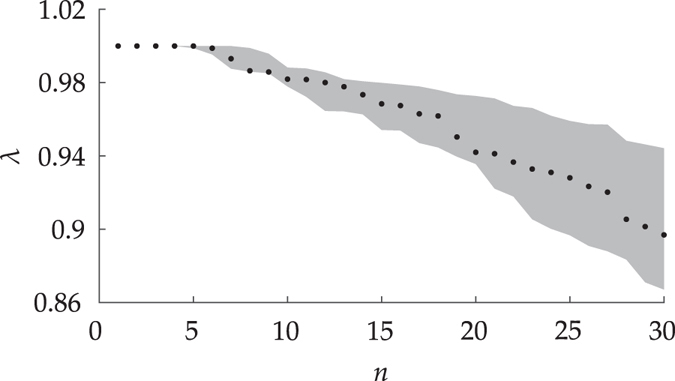
First 30 eigenvalues of the transition matrix *P* computed using two-day-long drifter trajectory data (dots) and uncertainties (gray shade) representing the total variation across eigenvalues computed from an ensemble of *P*s produced by randomly perturbing the trajectories. Computations and visualization carried using Matlab R2017a (http://www.mathworks.com/).

Left–right eigenvector pairs with *λ* > 0.97342 not discussed above do not provide any additional relevant information. Indeed, almost-invariant attractors and basins of attraction covering locations similar to those described above are in general revealed (a subset of these eigenvectors are shown in Fig. S4 of the Supplementary Information). Left–right eigenvector pairs with *λ* < 0.97342 can be ignored because of their lower relative statistical significance. Figure 5 shows a portion of the discrete eigenspectrum of *P* and an estimate of the associated uncertainty. Specifically, the dots correspond to the first 30 eigenvalues (out of a total of *N* = 1500). For the *n*th eigenvalue the width of the grey shading corresponds to the maximum variation across an ensemble of 100 realizations of the *n*th eigenvalue computed from transition matrices constructed using randomly perturbed drifter trajectories with an amplitude ranging from 500 m to 1.5 km, i.e., from at least as 100 times as large as the Global Positioning System (GPS) accuracy to up 10 times large as the *Argos* satellite system mean positioning error. Note that the uncertainty grows most noticeably starting at around the 15th eigenvalue, *λ* ≈ 0.96841, suggesting a cutoff for eigenvector analysis.

A timescale *τ* characterizing the level of invariance of a set can be calculated by thinking of *λ* < 1 as a decay rate. Let *T* denote the transition time between states of the Markov chain represented by the transition matrix *P*. Let *α* < 1 be the fraction by which a set has decayed under *n* applications of *P*. Then $\tau =\frac{\mathrm{log}\,\alpha }{\mathrm{log}\,\lambda }T$. Taking *α* = 0.5, representing a set’s “half life”, the invariance timescales of the attracting sets identified in the left column of Fig. 4 approximately are, from top to bottom, *τ* = 13 years, 3 years, 3 months, and 2 months. These invariance timescales are consistent with the accumulation persistence times roughly estimated above from the forward evolution of a uniform tracer distribution.

### Construction of the Lagrangian dynamical geography

**Figure 6:**
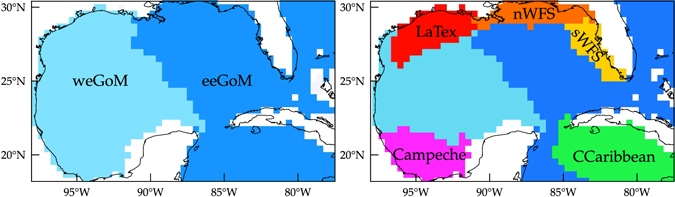
Main (left) and refined (right) dynamical geographies with weakly interacting provinces corresponding to domains of attraction associated to the largest almost-invariant attractors identified. Computations carried using Matlab R2017a (http://www.mathworks.com/) and visualization using Tecplot 360 2016 R2 (http://www.tecplot.com/).

With the knowledge acquired using the eigenvector method we can now move on constructing a Lagrangian dynamical geography for the eGoM. This is done by patching together the weakly communicating basins of attraction revealed through an appropriate thresholding of the right eigenvectors. We have found that setting the threshold at 0.005 gives satisfactory results; similar thresholds have been considered in earlier work^18^. The trivial partition has a single province covering virtually the whole eGoM. The coarsest nontrivial partition (Fig. 6, left panel) has two dynamical provinces separated by a nearly straight boundary connecting the Mississippi River Delta and the easternmost tip of the Yucatan Peninsula. The eastern eGoM province is labeled eeGoM, while the western eGoM province is denoted weGoM. A refined partition (Fig. 6, right panel) has five additional dynamical provinces roughly spanning the northern West Florida Shelf, the southern West Florida Shelf, the Louisiana–Texas Shelf, the Bay of Campeche, and a large region over the Cuban Caribbean Sea. These provinces are denoted nWFS, sWFS, LaTex, Campeche, and CCaribbean, respectively. Tracers initially within these provinces will spend more time moving and eventually temporarily accumulating in regions within them than dispersing across their boundaries. These dynamical provinces thereby set the way that distant regions in the eGoM, and the GoM in particular, are connected by tracer motion.

**Figure 7:**
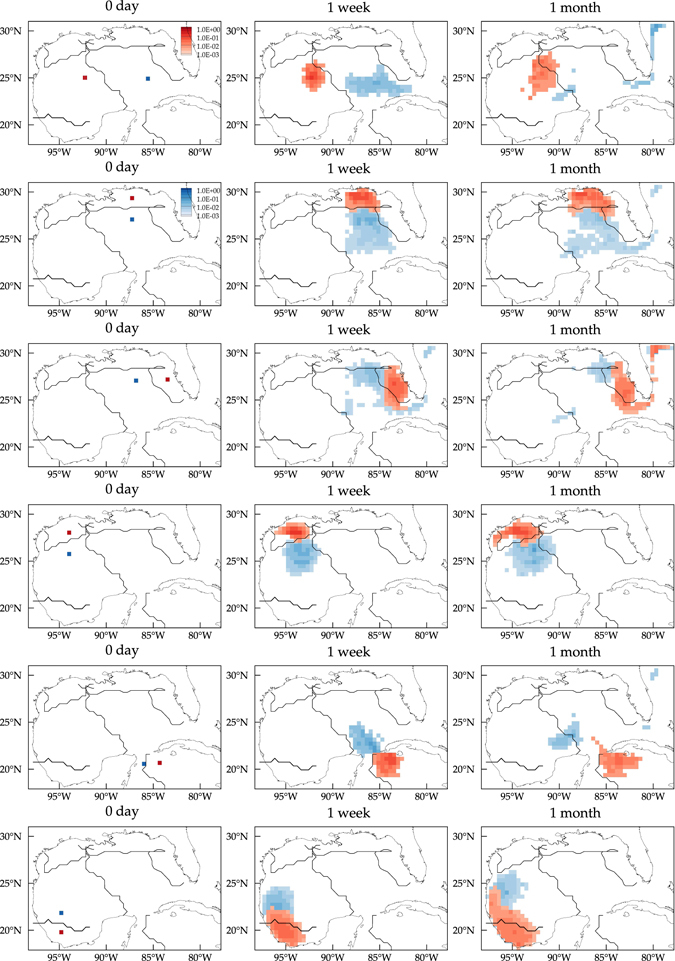
Forward evolution under the action of the transition matrix *P* of tracers released at various localized regions with dynamical geographic divisions overlaid. The tracer concentration is normalized by the mean concentration. Computations carried using Matlab R2017a (http://www.mathworks.com/) and visualization using Tecplot 360 2016 R2 (http://www.tecplot.com/).

To illustrate the impact of the Lagrangian dynamical geography on the Lagrangian circulation explicitly, forward tracer evolutions from various point sources are shown in Fig. 7. The source locations were chosen to straddle some of the dynamical province boundaries. Note that while tracers are released relatively nearby, they take on quite different paths. Note also that the level of leakage through the boundary of a dynamical province is influenced locally by the level of invariance of the attracting region contained within the province and remotely by that of any attractors outside of the province but sufficiently close to it. For instance, leakage through the border of the Campeche and LaTex provinces is larger than through the border between the weGoM and eeGoM provinces. This is consistent with the attracting sets contained inside the Campeche and LaTex provinces having a shorter invariance timescale (2 months) compared to the invariance timescale of the attracting region lying near the Perdido Foldbelt (3 years), which remotely affects the Lagrangian dynamics inside the Campeche and LaTex provinces.

**Figure 8:**
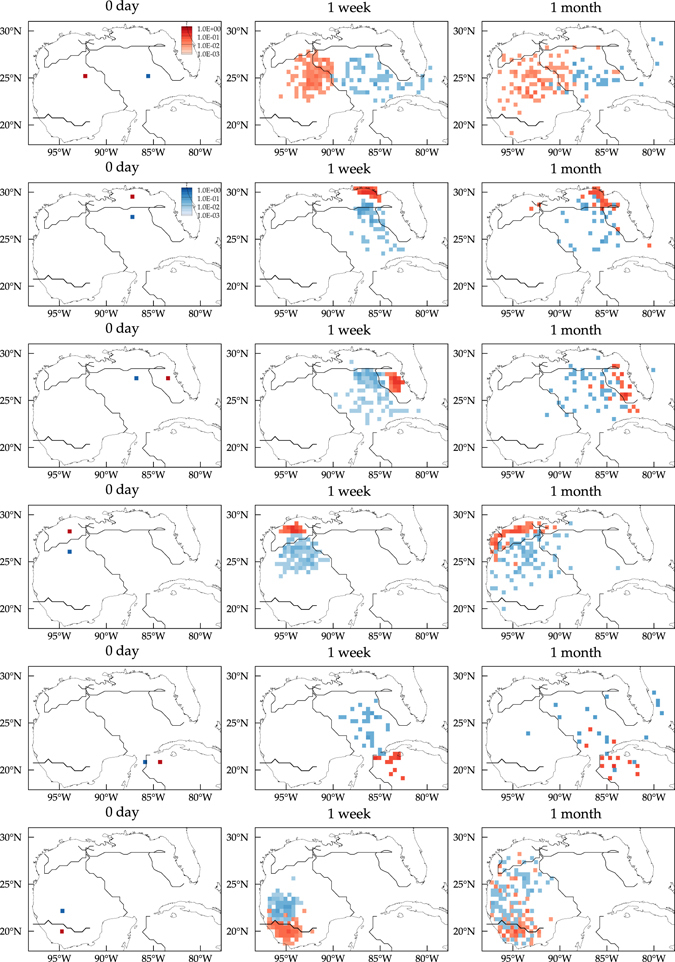
Evolution of normalized drifter densities for all drifters that have gone through the locations indicated in the left column at some point in time. Computations carried using Matlab R2017a (http://www.mathworks.com/) and visualization using Tecplot 360 2016 R2 (http://www.tecplot.com/).

A verification of the significance of the Lagrangian dynamical geography *independent* of the Markov model derived here is given in Fig. 8. This figure shows evolutions of positions of drifters, plotted as densities, for drifters going through the same tracer source locations as in Fig. 7. More specifically, to create these densities, for all drifters that have gone through these locations at some point in time, their positions were recorded after 7 days and 1 month, and the number of drifters falling in a given bin of of the grid in Fig. 1 counted and divided by the total number of drifters involved. Note that the drifter densities evolve in a way that is broadly consistent with the constraints imposed by the boundaries of the Lagrangian dynamical geography.

### Independent observational support for the geography

We close with an account of independent analyses of various different observations that provide additional reality checks to the eigenvector method results and thus the Lagrangian dynamical geography implied. Analysis of satellite altimetry data^31^ and ship-drift^12^ and drifter trajectory data^32^ with techniques different than those employed here have revealed the existence of a mean westward flow in the GoM. This provides a mechanism for accumulation on western side of the GoM, and hence independent sustain for the almost-invariant attracting set the eigenvector method detected near the Perdido Foldbelt. The persistent cyclonic gyres south of Cuba observed in drifter trajectory data^33^ provide a mechanism for temporary retention in the region, and hence support for the almost-invariant attracting set identified there. Satellite-tracked drifter trajectories reveal persistent cyclonic motion in Bay of Campeche^13, 14^, providing too a mechanism for temporary retention consistent with our findings. In turn, Yang *et al*.^11^ noted that satellite-tracked drifters deployed on the northern GoM tend avoid the southern West Florida Shelf, calling this region a “forbidden zone”. Such a forbidden zone, whose boundary was later characterized using nonlinear dynamics techniques^34, 35^, forms one of the provinces of our dynamical geography. Analyzing card and bottle landings over 1955–1987 as well as marine mammals and turtle strandings, Lugo-Fernández *et al*.^36^ reported coastal areas acting as attractors in near coincidental position with some of those identified by the left eigenvectors of the transition matrix constructed here. Furthermore, these authors added “… the surface currents from ship-drift suggest that the Gulf [of Mexico] may be bisected by a line along 86° or 87°W to the Mississippi Delta”. This is consistent with the minimal partition of the eGoM resulting from the application of the eigenvector method.

## Conclusions

Using an unprecedentedly large collection of satellite-tracked surface drifter trajectory data in the GoM and its vicinity, we constructed a Markov-chain representation of the GoM’s surface-ocean Lagrangian dynamics. Analyzing the eigenvectors of the transition matrix of the chain we identified almost-invariant regions of attraction and their basins of attraction. With this information we then constructed a Lagrangian dynamical geography with weakly interacting provinces. Constraining the connectivity between distant places in the GoM, the dynamical geography constructed can have implications for offshore oil exploration, oil spill contingency planning, pollution mitigation, and fish larval connectivity assessment among many other activities of practical interest.

Our results were found to be robust under arbitrary data reductions, but biases due to irregular spatiotemporal sampling may still be possible. While some drifters in the database cover extended periods of time, representing quite well all possible dynamical scenarios, a large number of drifters were deployed in localized regions in the northern GoM, sampling particular dynamical conditions observed during the months over which the experiments lasted. Other drifters targeted particular dynamical features like Loop Current rings, potentially biasing accumulation on the western GoM. Currently underway is an assessment of the importance of these potential biases using synthetic drifter trajectories as generated by different ocean general circulation models.

## Methods

### Eigenvector method

The eigenvector method^18^ employed here is rooted in Markov-chain theory concepts that have previously been used to approximate invariant sets in dynamical systems using short-run trajectories^15–17^. The dynamical system of interest is that governing the motion of fluid particles, which are described by satellite-tracked drifters on the ocean surface.

Let *X* be a closed flow domain on the plane and denote by *T*(*x*) the end point of a trajectory starting at *x* ∈ *X* after some short time. A discretization of the dynamics can then be attained using Ulam’s method^30, 37^ by dividing the domain *X* into *N* connected bins $\{{B}_{1},\ldots ,{B}_{N}\}$, which will here be assumed of the same area for simplicity. The proportion of mass in *B*_*i*_ mapped to *B*_*j*_ under one application of *T* is (approximately) equal to

1 $${P}_{ij}=\frac{\#\,{\rm{of}}\,{\rm{particles}}\,{\rm{in}}\,{B}_{i}\,{\rm{that}}\,{\rm{are}}\,{\rm{mapped}}\,{\rm{to}}\,{B}_{j}}{\#\,{\rm{of}}\,{\rm{particles}}\,{\rm{in}}\,{B}_{i}}.$$

The *transition matrix P* defines a Markov-chain representation of the dynamics, with the entries *P*_*ij*_ equal to the conditional transition probabilities between bins, representing the states of the chain. Let ${\bf{f}}=({f}_{1}\cdots {f}_{N})$ be the discrete representation of *f*(*x*), the probability density of some tracer distribution. Its forward evolution is calculated under right multiplication by *P*, i.e.,

2 $${{\bf{f}}}^{(k)}={\bf{f}}{P}^{k},\quad k=1,2,\ldots .$$

The above is the discrete representation of the push forward of *f*(*x*) under the action of the flow map *T*, namely, ${\mathscr{P}}\,f(x)=f\circ {T}^{-1}(x)\,|{\rm{\det }}\,{\rm{D}}{T}^{-1}(x)|$, where ${\mathscr{P}}$ is called a *transfer* or *Perron–Frobenius operator* ^17^. If the flow is incompressible, in which case det D*T*(*x*) = 1, then ${\mathscr{P}}\,f(x)$ corresponds to an area-preserving rearrangement of *f*(*x*).

Assume that *P* is *regular*, i.e., there exists *N* < ∞ such that (*P* ^*n*^)_*ij*_ > 0 for all *n* ≥ *N* and all 1 ≤ *i*, *j* ≤ *N*. This means that *P* is both *irreducible* (i.e., for each 1 ≤ *i*, *j* ≤ *N*, ${({P}^{{n}_{ij}})}_{ij} > 0$ for some *n*_*ij*_ < ∞, so all states communicate) and *aperiodic* (i.e., for each 1 ≤ *i* ≤ *N* there exists *M* < ∞ such that (*P* ^*m*^)_*ii*_ > 0 for all *m* ≥ *M*, so no state occurs recurrently). By the Perron–Frobenius theorem, the eigenvalue spectrum of *P* satisfies |*λ*| ≤ 1. The eigenvalue *λ* = 1 is simple and the corresponding left and right eigenvectors both are positive. Furthermore, there exists a unique limiting distribution, given by

3 $${f}_{i}^{(\infty )}={({P}^{\infty })}_{ij} > 0\,\forall j,$$

which satisfies

4 $${{\bf{f}}}^{(\infty )}={{\bf{f}}}^{(\infty )}P.$$

Note that the limiting distribution is an invariant distribution (it does not change under the action of *P*). Moreover, it is a left eigenvector of *P* with eigenvalue *λ* = 1. The associated right eigenvector is ${\bf{1}}={(1\cdots 1)}^{{\rm{{\rm T}}}}$, as required by row-stochasticity of *P*, viz.,

5 $$P{\bf{1}}={\bf{1}}.$$

When *P* is not regular, no unique limiting distribution exists, and a nontrivial connection between left and right eigenvectors of *P* with invariant attracting sets and basins of attraction, respectively, can be established.

To illustrate the above, consider the reduced chain comprising 5 states {*A*, *B*, *C*, *D*, *E*} represented by

6 $$P=\begin{array}{cc} & \begin{array}{ccccc}\,A\,\, & B\,\, & C\,\, & D\,\, & E\,\,\end{array}\\ \begin{array}{c}A\\ B\\ C\\ D\\ E\end{array} & (\begin{array}{ccccc}0.8 & 0.2 & 0 & 0 & 0\\ 0.3 & 0.7 & 0 & 0 & 0\\ 0 & 0.2 & 0.8 & 0 & 0\\ 0 & 0 & 0 & 0.7 & 0.3\\ 0 & 0 & 0 & 0 & 1\end{array})\end{array}$$

**Figure 9:**
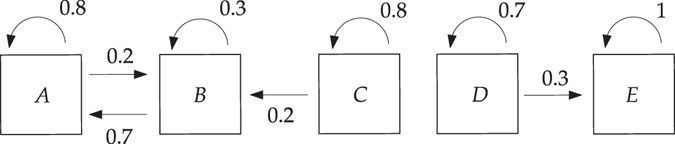
Reduced Markov chain comprising 5 states with transition probabilities of moving among states indicated which is used to illustrate the idea of the eigenvector method. Figure generated using Ipe 7.2.7 (http://ipe.otfried.org/).

The chain with the transition probabilities among pairs of states and corresponding directions indicated is sketched in Fig. 9.

States {*A*, *B*, *C*} do not communicate with states {*D*, *E*} and thus the chain is not irreducible and therefore not regular. In particular, classes {*A*, *B*} and {*E*} are closed (*P* = 0 for entries linking states in and outside of the class) and furthermore absorbing (*P* > 0 for at least one entry linking a state outside of the class with one in the inside thereof). This implies the existence of two limiting distributions, one supported on states {*A*, *B*} and another one supported on state {*E*}, which are reached from any distributions initially supported on their basins of attraction, given by states {*A*, *B*, *C*} and {*D*, *E*}, respectively. Direct computation gives ${{\bf{f}}}_{AB}^{(\infty )}=(\frac{7}{9}\frac{2}{9}000)$ and ${{\bf{f}}}_{E}^{(\infty )}=\mathrm{(00001)}$, respectively. Linear combinations $a{{\bf{f}}}_{AB}^{(\infty )}+b{{\bf{f}}}_{E}^{(\infty )}$, $a,\,b\ne 0$, also are limiting distributions, which are reached from arbitrary distributions with support on {*A*, *B*, *C*} and {*D*, *E*}.

Now, because the chain is not regular, the largest eigenvalue of *P*, *λ* = 1, is not simple and in this case has multiplicity 2. The left eigenvectors with *λ* = 1 are given by limiting distributions ${{\bf{f}}}_{AB}^{(\infty )}$ and ${{\bf{f}}}_{E}^{(\infty )}$. The corresponding right eigenvectors are given by ${\mathrm{(11100)}}^{{\rm{{\rm T}}}}$ and ${\mathrm{(00011)}}^{{\rm{{\rm T}}}}$, which are supported on the basins of attraction of these limiting distributions; cf. Norris^28^, thm. 1. 2. 3 or Froyland *et al*.^18^, thm. 1.

The above illustrates the essence of the eigenvector method: invariant attracting sets and disjoint basins of attraction can be revealed from the structure of the left and right eigenvectors of *P* with eigenvalue 1, respectively. This motivates the possibility of revealing almost-invariant attracting sets and weakly disjoint basins of attraction, relevant in dynamical systems governing motion that exhibits transient behavior, by inspecting left and right eigenvectors of *P* with eigenvalues close to 1.

### Data

The analysis was performed using a large collection of drifter trajectory data over 1994 through 2016. A total of 3207 drifter trajectories from several different sources were considered. Specifically, 1002 quarter-hourly trajectories of drifters deployed during the LAgrangian Submesoscale ExpeRiment (LASER); 575 quarter-daily trajectories from the National Oceanic and Atmospheric Administration (NOAA) Global Drifter Program (GDP); 523 daily trajectories from the Surface Current Lagrangian-Drift Program (SCULP); 441 hourly trajectories from the Horizon Marine Inc.’s Eddy Watch program; 372 trajectories from the Centro de Investigación Cientfica y de Educación Superior de Ensenada (CICESE)–Petróleos Mexicanos (Pemex) “Caracterización Metoceánica del Golfo de México”; 302 quarter-hourly trajectories from the Grand LAgrangian Experiment (GLAD); 68 quarter-hourly from the NOAA/Atlantic Oceanic and Meteorological Laboratory (AOML) South Floida Program (SFP); and 30 hourly drifters recorded by the U.S. Coast Guard (UCSC) during LASER.

The LASER experiment used biodegradable drifters designed by researchers of the Gulf of Mexico Research Initiative’s Consortium for Advanced Research on Transport of Hydrocarbon in the Environment (CARTHE) at the University of Miami’s Rosenstiel School of Marine and Atmospheric Science. They consisted of two main parts, a surface wheel-shape float of 38-cm diameter and a cross-shape drogue made of 38-cm × 38-cm rigid panels, both connected by a 15-cm flexible tether. The positions of the drifters were tracked by GPS. The GDP drifters^38^ follow the SVP (Surface Velocity Program) design^39^, consisting of a spherical float which is drogued with a holey sock at 15 m. Their positions are tracked by the *Argos* system or GPS. The drifters from SCULP^40, 41^ followed the Coastal Ocean Dynamics Experiment (CODE) design^42^. These drifters consist of a 1-m-long rigid tube with 4 sails attached forming a cross; floatation is supplied by small floats fasten to the sails. Their positions were tracked using the *Argos* system. The drifters from the EddyWatch^®^ program and the CICESE–Pemex project are Far Horizon Drifters (FHD), manufactured by Horizon Marine Inc.^43, 44^. They consist of a cylindrical buoy attached to a 45-m tether line, attached itself to a 1.2-m “para-drogue”. These instruments are deployed by air, so the drogue serves both as parachute to protect the buoy when air deployed, as well as to reduce wind slippage of the buoy as it drifts in the water. Positions are tracked using GPS. Most drifters from GLAD^3, 4, 6^ were CODE type and GPS tracked. Finally, the SPF and USCG drifters are also CODE-type drifters with positions tracked by the *Argos* system.

For the purposes of the present work, which focuses on long-time behavior, it was sufficient to consider daily trajectories, which required us to subsample those trajectories recorded at a higher than daily rate.

## Electronic supplementary material

Supplementary Information
