# Supplementary material for: Lagrangian dynamical geography of the Gulf of Mexico
Source: Sci Rep. 2017 Aug 1;7:7021. doi: 10.1038/s41598-017-07177-w (PMC5539163; doi:10.1038/s41598-017-07177-w)
Supplement: Supplementary file 1 — Supplementary Information [file 41598_2017_7177_MOESM1_ESM.pdf]

Supplementary Information for “Lagrangian dynamical Geography of the Gulf of Mexico”

P. Miron[1,*], F. J. Beron-Vera[1], M. J. Olascoaga[2], J. Sheinbaum[3], P. Pérez-Brunius[3], and G. Froyland[4]

[1]Department of Atmospheric Sciences, Rosenstiel School of Marine and Atmospheric Science, University of Miami, Miami, Florida, USA
[2]Department of Ocean Sciences, Rosenstiel School of Marine and Atmospheric Science, University of Miami, Miami, Florida, USA
[3]Departamento de Oceanografía Física, Centro de Investigación Científica y Educación Superior de Ensenada, Ensenada, Baja California, Mexico
[4]School of Mathematics and Statistics, University of New South Wales, Sydney, Australia
[*]pmiron@rsmas.miami.edu

Appendix A: Robustness of the results

The robustness of the drifter-data-based Markov chain representation of the GoM's Lagrangian dynamics is tested here under variations of the transition time (i.e., the length of the trajectory pieces used to compute the transition matrix) and reductions of the number of trajectories involved.

To evaluate the effect of the transition time, we compare the density distribution after 2000 days, starting from a uniform density in the eGoM. The resulting distributions using different transition times are presented in the top row of Fig. S1. The tracer amplification factor (TAF) is defined by:

$$\mathrm{TAF}(i) = c(i)\, N/C \tag{1}$$

where $c(i)$ is the local bin density, C is the total density initially inside the domain and N is the total number of bins. An average TAF value of 0.4 on the western side of the GoM indicates that after 2000 days, 40% of the density released is still located in the area. Visually the three approximations are very similar, and this is confirmed in the bottom row of Fig. S1 where the absolute error between each approximation is plotted. The error range $[0, 0.00126]$ is two orders of magnitude lower than the density range $[0, 0.435]$. For the selected spatial resolution, this proves the low impact of the transition time on the results. The influence starts to be noticeable when the transition time is selected over 6 weeks, mainly because trajectories are short and the resulting database is too thin to extract all the underlying dynamics of the GoM.

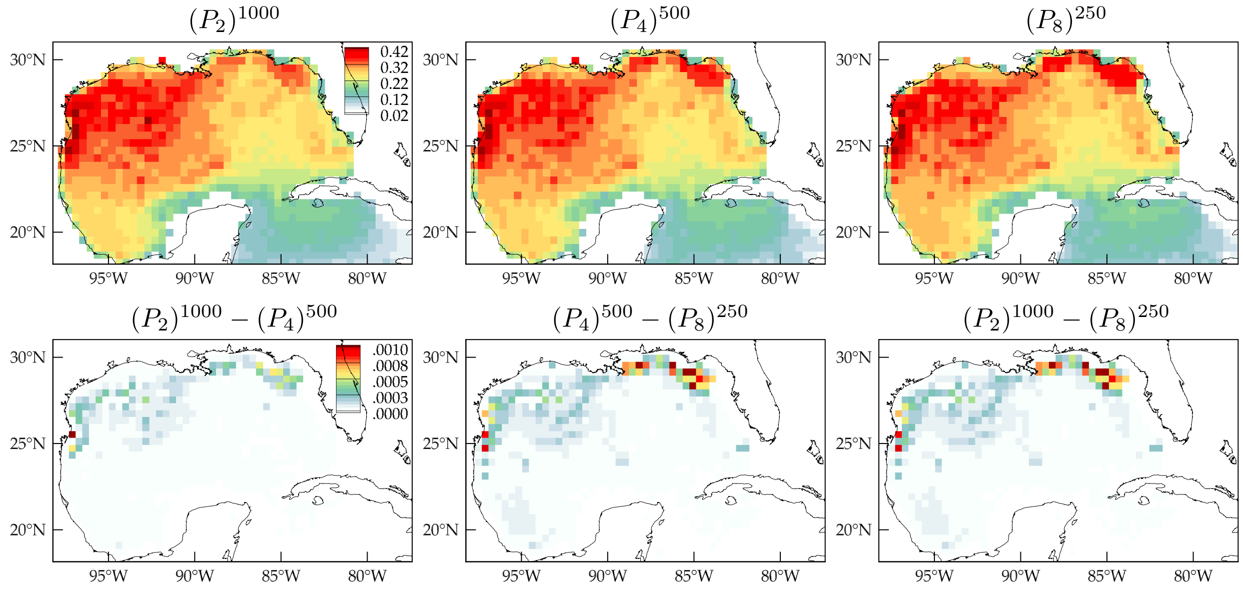

Figure S1. (top) Distribution on day 2000 of an initially uniform tracer density according to transition matrices constructed using on 2 (P_2), 4 (P_4), and 8 (P_8) day long trajectories. (bottom) Absolute difference between distributions. Computations carried using Matlab R2017a (http://www.mathworks.com/) and visualization using Tecplot 360 2016 R2 (http://www.tecplot.com/).

To further evaluate the effect of data reduction, the eigenvector analysis is performed with only a subset of the available drifter trajectories. Each row of Fig. S2 shows three different left eigenvectors obtained using 70% (top row) and 50% (bottom row) of the available data. While the eigenvalue magnitude are slightly different, the almost-invariant structures highlighted by the eigenvector distributions are very similar.

Appendix B: Additional supporting figures

Additional figures are included in this appendix showing the eigenvectors with eigenvalue on the unit circle (Fig. S3) and a subset of eigenvectors with eigenvalue close to unity different than those presented in Fig. 4 (Fig. S4).

The eigenvalue $\lambda = 1$ of P is the largest and further has multiplicity 4, consistent with the existence of 4 closed communicating classes in the Markov chain. The associated left eigenvectors (Fig. S3, left column) of P vanish everywhere over the eGoM except inside the regions occupied by these classes, where they take positive values.

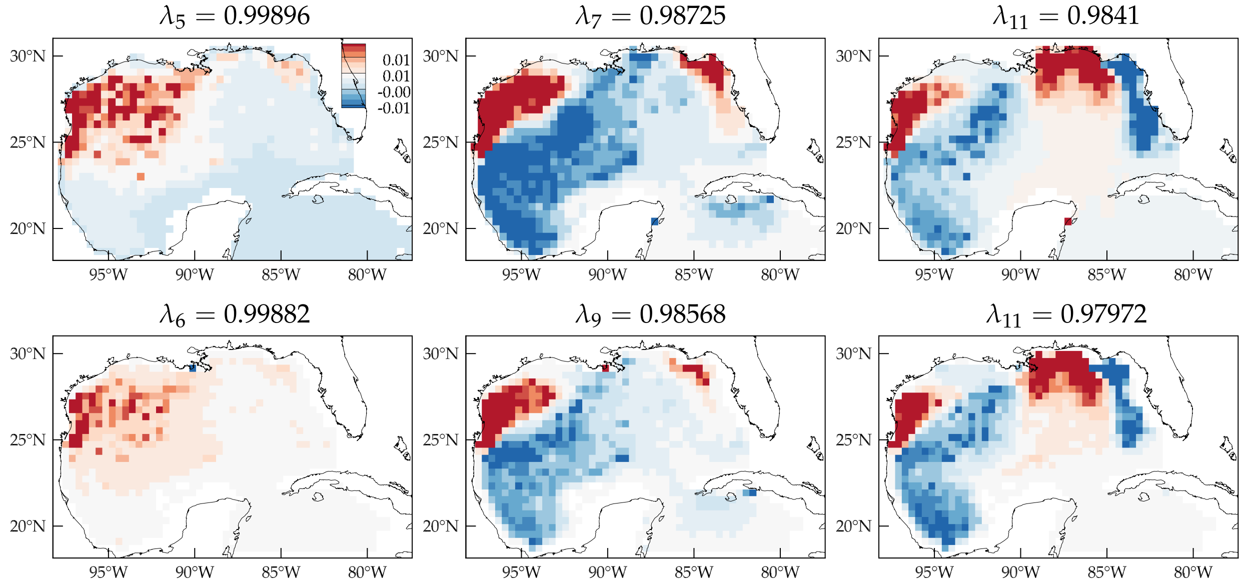

Figure S2. To validate the robustness of the method under data reductions, left eigenvectors (left) showing the locations of almost-invariant regions of forward-time attraction using 70% (top row) and 50% (bottom row) of the available drifter trajectories. Computations carried using Matlab R2017a (http://www.mathworks.com/) and visualization using Tecplot 360 2016 R2 (http://www.tecplot.com/).

The distributions defined by the left eigenvectors are invariant as they do not change under the action P. For the nonabsorbing classes, the $\lambda = 1$ right eigenvectors of P (Fig. S3, second and third rows in the right column) vanish everywhere over the eGoM except within the small regions where these classes are located, where they take positive constant values. For the absorbing classes, the right eigenvectors (Fig. S3, first and fourth rows in the right column) take positive constant values inside nonoverlapping domains containing the small regions where these classes are located and vanish outside. The domain including the absorbing class on the Bahamas Bank is small, tightly covering this class. The domain including the absorbing class on the western coast of the Florida Peninsula is large, spanning the entire eGoM except the other domain and the regions where the closed classes are located. Such nonoverlapping domains are the basins of attraction for the small absorbing classes, where the limiting invariant distributions are supported, as we have verified by the forward evolution of a uniform tracer. As we have already noted, neither these small attractors nor the small closed classes represent any relevant aspect of the Lagrangian dynamics other than mere beaching.

Appendix C: Additional supporting material

Numerical algorithm The eigenvector analysis does not require high computing resources. All the calculations were performed using MATLAB (http://www.mathworks.com/) and took less than a minute to complete on a regular desktop computer. The general algorithm is straightforward and summarize by the following steps:

1. Select a transition time (T) and a spatial resolution (Δx).
2. Split the domain (in our case the Gulf of Mexico) into square boxes (bins) of size $(\Delta x)^2$ delimiting the computational domain, see Fig. 1. For efficient location search, a structured grid initially cover a rectangular limiting area and then using the coastline data, the boxes outside of the Gulf limits are removed.
3. For each box B_i, find all particle ID and time pairs (ID,t) for which the particle ID at time t is in box B_i, and call this collection X_i.
4. Evolve all particles in X_i for T days. Call this collection Y_i.
5. For each box B_i and each box B_j, find those evolved particles in Y_i that lie in box B_j. Call this collection Y_{ij}.
6. Calculate P_{ij} using (Eq. 1) as $|Y_{ij}|/|Z_i|$.

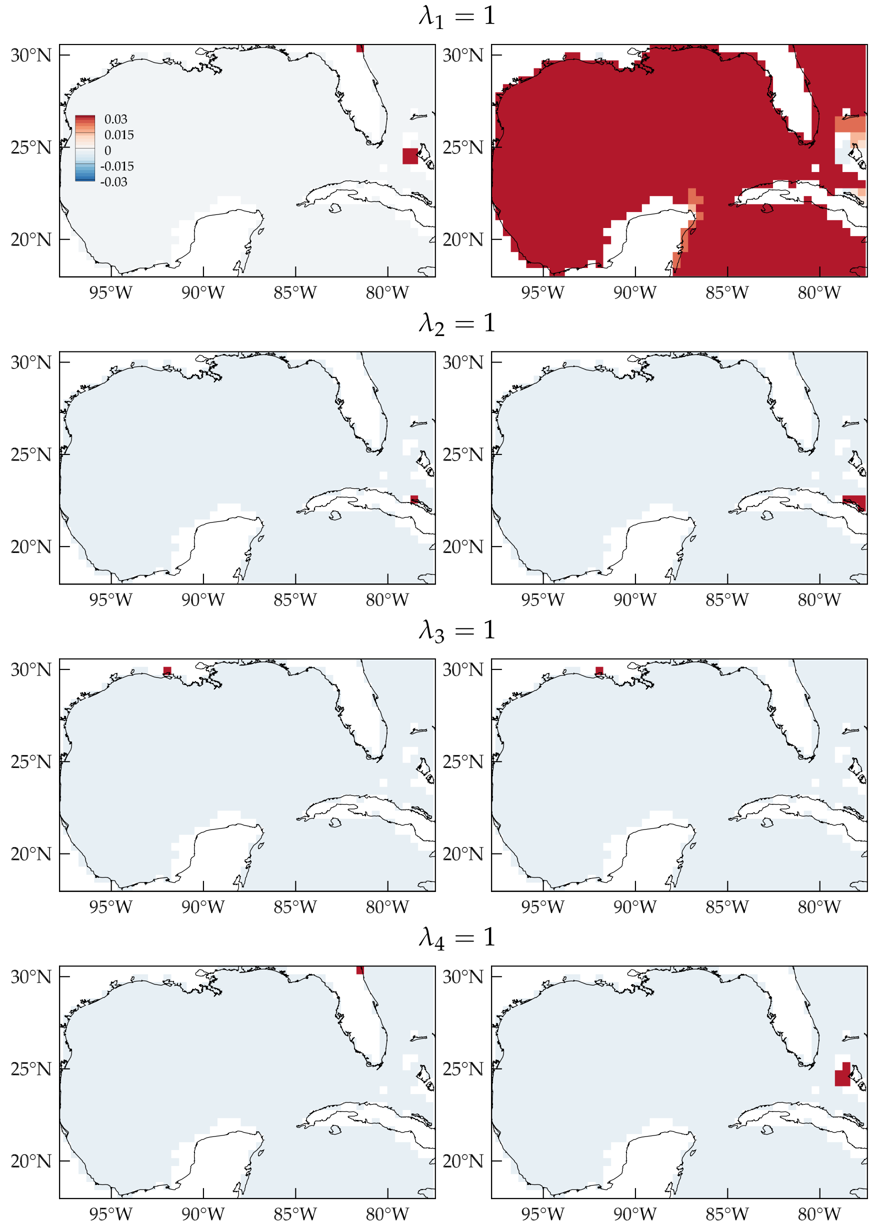

Figure S3. Left (left column) and right (right column) eigenvector fields with eigenvalues on the unit circle. Computations carried using Matlab R2017a (http://www.mathworks.com/) and visualization using Tecplot 360 2016 R2 (http://www.tecplot.com/).

7. Calculate eigenvalues, left and right eigenvectors of the transition matrix P.

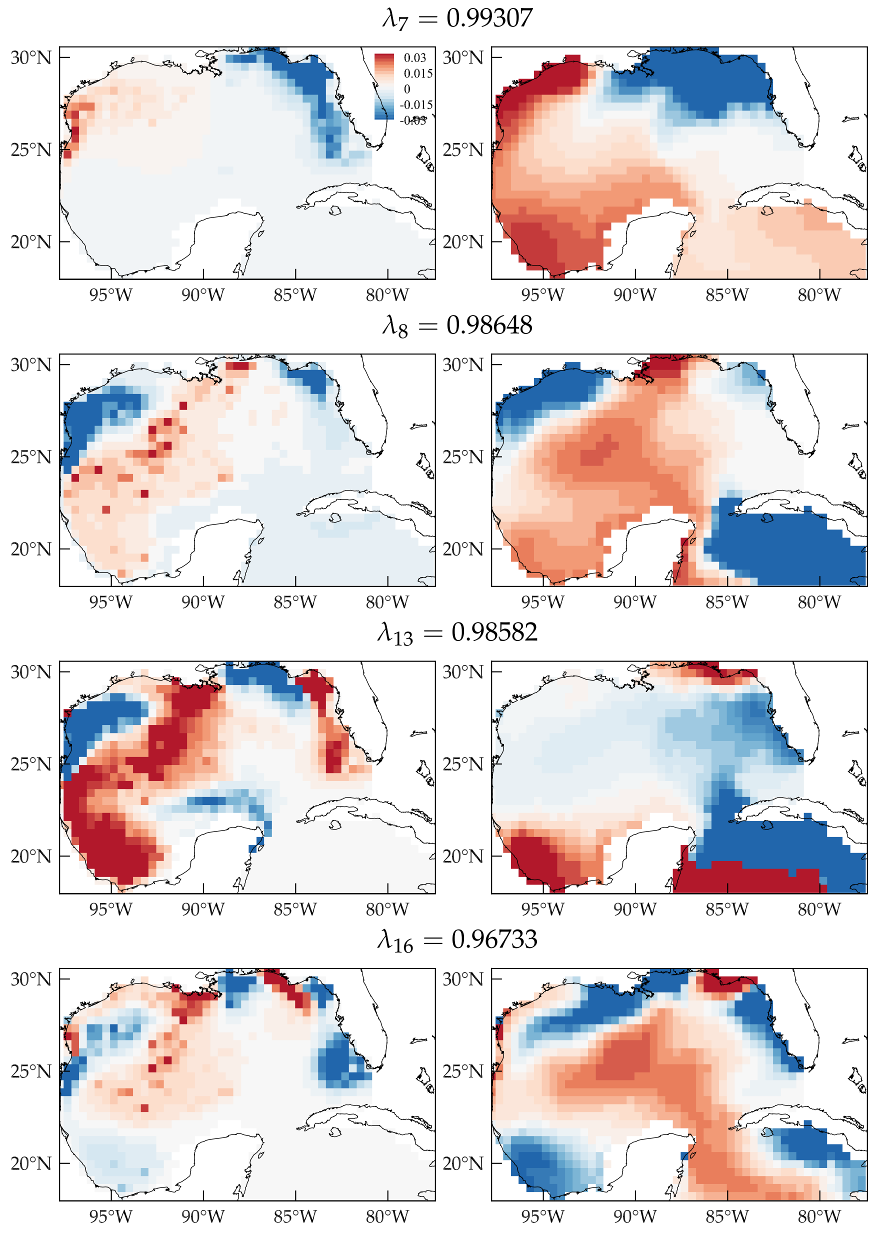

Figure S4. As in Fig. 4, but for some additional eigenvectors, which reveal almost-invariant regions of attraction and basins of attraction similar to those already identified. Computations carried using Matlab R2017a (http://www.mathworks.com/) and visualization using Tecplot 360 2016 R2 (http://www.tecplot.com/).
